# Short-Term Exercise Training Inconsistently Influences Basal Testosterone in Older Men: A Systematic Review and Meta-Analysis

**DOI:** 10.3389/fphys.2018.01878

**Published:** 2019-01-14

**Authors:** Lawrence D. Hayes, Bradley T. Elliott

**Affiliations:** ^1^Active Ageing Research Group, Department of Medical and Sport Sciences, University of Cumbria, Lancaster, United Kingdom; ^2^Translational Physiology Research Group, School of Life Sciences, University of Westminster, London, United Kingdom

**Keywords:** endurance, endocrine, exercise, HIIT, interval, resistance, testosterone, weight training

## Abstract

**Background:** The age-associated decrease in testosterone is one mechanism suggested to accelerate the aging process in males. Therefore, approaches to increase endogenous testosterone may be of benefit. The aim of this paper was to undertake a Preferred Reporting Items for Systematic Reviews and Meta-Analysis (PRISMA)-accordant meta-analysis concerning the effect of exercise on total (TT), bioavailable (bio-T), free (free-T), and salivary (sal-T) testosterone in older males.

**Methods:** Databases were searched up to and including 20th February 2018 for the terms “testosterone AND exercise AND aging AND males,” “testosterone AND exercise AND old AND males,” “testosterone AND training AND aging AND males,” and “testosterone AND training AND old AND males”. From 1259 originally identified titles, 22 studies (randomized controlled trials; RCTs; *n* = 9, and uncontrolled trials; UCTs; *n* = 13) were included which had a training component, participants ≥60 years of age, and salivary or serum testosterone as an outcome measure. Meta-analyses were conducted on change to testosterone following training using standardized difference in means (SDM) and random effects models.

**Results:** The overall SDM for endurance training, resistance training, and interval training was 0.398 (95% CI = 0.034–0.761; *P* = 0.010), −0.003 (95% CI = −0.330–0.324; *P* = 0.986), and 0.283 (95% CI = 0.030–0.535; *P* = 0.028), respectively. Resistance training exhibited a qualitative effect of hormone fraction whereby free-T resulted in the greatest SDM (0.253; 95% CI = −0.043–0.549; *P* = 0.094), followed by TT (0.028; 95% CI = −0.204–0.260; *P* = 0.813), and resistance training negatively influenced bio-T (−0.373; 95% CI = −0.789–0.042; *P* = 0.078). Due to the small number of studies, subgroup analysis was not possible for endurance training and interval training studies.

**Conclusions:** Data from the present investigation suggests that resistance training does not significantly influence basal testosterone in older men. Magnitude of effect was influenced by hormone fraction, even within the same investigation. Aerobic training and interval training did result in small, significant increases in basal testosterone. The magnitude of effect is small but the existing data are encouraging and may be an avenue for further research.

## Introduction

### Rationale

Muscle mass and function are particularly important in older adults, as epidemiological evidence suggests a positive relationship with longevity (Metter et al., 2004; Srikanthan and Karlamangla, 2014). Sarcopenia, defined as a loss of muscle mass coupled with functional deterioration, is now a clinically recognized disease deserving international attention (Cao and Morley, 2016). Given the increasing age of the world's population, maintaining muscle function into later life is imperative to avoid spiraling public health costs. Like muscle mass and function, serum testosterone typically declines with age (Tenover, 1997; Harman et al., 2001), and low testosterone is associated with many non-communicable diseases such as diabetes (El Baba and Azar, 2013; Mazur et al., 2014), cardiovascular disease (Schooling, 2014; Yeap, 2015), Alzheimer's disease (Lv et al., 2016), dementia (Carcaillon et al., 2014), obesity (Kelly and Jones, 2015), and ultimately mortality (Shores et al., 2012; Muraleedharan et al., 2013; Yeap, 2015). Several studies have reported improved health outcomes with exogenous testosterone administration, but side-effects are common, particularly cardiovascular events (Kim, 1999; Basaria et al., 2010; Yeap, 2015). Some effects of testosterone administration mimic those of exercise training. For example, Atkinson et al. (2010) reported testosterone administration preserved muscle mass in elderly individuals, which is the same effect appropriately prescribed exercise exerts (Frontera et al., 1988; Fiatarone et al., 1990; Herbert et al., 2017b).

In view of the complications with testosterone administration, exercise has been proposed as a non-pharmacological intervention to increase serum testosterone in older males (Swerdloff and Anawalt, 2014; Hayes et al., 2015d). However, the effect of exercise on testosterone is poorly defined, even within the same research group. For example, recent data suggest that although endurance-trained masters athletes and sedentary older adults exhibit similar total testosterone (TT), bioavailable testosterone (bio-T), and free testosterone (free-T) (Hayes et al., 2015b), salivary testosterone (sal-T) significantly differed between the trained and untrained older men (Hayes et al., 2013a). Further, although no difference in mean TT existed, more of the sedentary individuals were classed as biochemically hypogonadal (clinically low TT) than the masters athletes (Hayes et al., 2017b). In contrast, Cooper et al. (1998) noted masters endurance runners had greater TT concentrations (~19 nmol·L^−1^ vs. ~15 nmol·L^−1^), but lower free androgen index (an estimate of biologically active testosterone; ~21 vs. ~31) than sedentary counterparts. However, the clinical significance of greater basal testosterone, within a “normal” physiological range, is unknown.

Different exercise modalities (i.e., endurance training, resistance training, interval training), and within-mode variables (i.e., intensity, volume, duration) may cause further discrepancies between investigations. Moreover, the portion of testosterone measured is not consistent between studies and can influence the direction and magnitude of response to exercise (Hayes et al., 2015d). For clarity, serum testosterone is mainly bound to sex hormone binding globulin (SHBG) and albumin. SHBG-bound testosterone is unavailable tissue uptake, whereas albumin-bound testosterone has access to target tissues because albumin-bound testosterone dissociates rapidly (Vermeulen et al., 1999). The non-SHBG-bound testosterone bound to albumin is therefore referred to as “bioavailable.” The portion of testosterone completely unbound to SHBG or albumin is referred to as “free.” TT encompasses SHBG-bound, bioavailable (albumin-bound), and unbound testosterone (i.e., 100% of that measured in the blood). Although these definitions are commonly used, there are numerous analytical methods for direct detection of testosterone, but also direct and indirect methods for quantifying bio-T and free-T. Different methods of analysis have dissimilar levels of variance and precision, which may conflate results. Furthermore, numerous exercise studies now measure testosterone in saliva (Keevil et al., 2017; Arruda et al., 2018; Chen et al., 2018) because of the ease of sample collection, despite methodological concerns (Hayes et al., 2015a,c, 2016). As such, the following normative values and clinical thresholds values cannot be applied to all laboratories, and must be interpreted with caution. Ranges for TT have been outlined as 10.4–32.6 nmol·L^−1^ (300–940 ng·dl^−1^) for 30 year old males, 9.3–31.3 nmol·L^−1^ (268–903 ng·dl^−1^) for 50 year old males, and 8.6–30.7 nmol·L^−1^ (248–885 ng·dl^−1^) for 70 year old males (Bjerner et al., 2009), with Harman et al. (2001) suggesting a threshold of 11.3 nmol·L^−1^ (326 ng·dl^−1^) for clinically low testosterone. However, Lazarou et al. (2006) reported that there were 17 different threshold values for TT to define hypogonadism across 25 laboratories. Indeed, the threshold for hypogonadism diagnosis varied by 350% (130 ng·dl^−1^-450 ng·dl^−1^ (4.5–15.6 nmol·L^−1^).

### Objectives

Despite the potential of exercise training to increase testosterone in older males, there was no meta-analysis to provide pooled analysis of published studies to date. Therefore, the aim of this investigation was to conduct meta-analyses on the effect of aerobic, resistance, and interval training on TT, bio-T, free-T, and sal-T. A secondary aim was to investigate study characteristics (i.e., research design and hormone fraction reported) on magnitude of effect.

## Methods

### Eligibility Criteria

This meta-analysis was conducted according to the Preferred Reporting Items for Systematic Reviews and Meta-Analysis (PRISMA) guidelines. Studies that met the following criteria were included: (1) published as a full-text manuscript; (2) not a review; (3) participants were apparently healthy older males (mean group age ≥60 years); (4) studies were required to employ an intervention design and include an exercise training period of >4 weeks. Additionally, descriptive data (e.g., sample size, mean, and standard deviation) were required to be reported. Where this was not possible, details were requested from authors. The primary aim was to investigate whether basal testosterone was affected by exercise training and therefore we only included studies that measured testosterone (TT, bio-T, free-T, or sal-T). Where an investigation took multiple measures, we included them as separate datasets.

Initially, this review aimed to consider randomized controlled trials (RCTs) and non-randomized control trials (CTs). However, due to the small number of RCTs and CTs, the study was extended to include uncontrolled trials (UCTs). For clarity, UCTs were analyzed separately from RCTs and CTs.

### Information Sources

PubMed, ScienceDirect, and SPORTDiscus were searched with no start data, up until 20th February 2018. The search was performed within all fields and terms were “testosterone AND exercise AND aging AND males,” “testosterone AND exercise AND old AND males,” “testosterone AND training AND aging AND males,” and “testosterone AND training AND old AND males.”

### Study Selection

Both authors conducted the eligibility assessment in an unblinded and standardized manner. Once each database search was completed and manuscripts were sourced, all studies were downloaded into a single reference list with duplicates removed. Titles and abstracts were then screened for eligibility and full texts were only retrieved for studies with testosterone and an exercise intervention incorporated. Two independent reviewers then read and coded all the included articles using the PEDro scale (Maher et al., 2003). Full texts were then thoroughly assessed using the complete eligibility criteria with first and second authors confirming inclusion and exclusion. Following this quality assessment, the same reviewers read and coded each of the studies and assessed the following moderators: design method (RCT or UCT), exercise type (endurance training, resistance training, or interval training), and hormone fraction (total, free, bioavailable, salivary). Furthermore, participant descriptions and training programme variables were extracted with as much detail provided by the authors. Any disagreement between both reviewers was discussed in a consensus meeting, and unresolved items were addressed by a third independent reviewer for resolution.

### Data Collection Process

Data were extracted for pre- and post-training basal testosterone concentrations. In cases of missing data, authors were contacted via email and asked to provide necessary information. If no response was received, means and standard deviations (SDs) were estimated from figures using computer software (Image J, Maryland, USA, Imagej.net). Information was imported into a spreadsheet, which was specifically designed for meta-analyses (Comprehensive meta-analysis, NJ, USA).

### Data Items

Heterogeneity was quantified with the *I*^2^ statistic. An *I*^2^ value of 25% may be interpreted as low, 50% as moderate and 75% as high between study heterogeneity. Three random-effect meta-analyses (endurance training, resistance training, and interval training) were conducted as each of these training types have different physiological demands and subsequent adaptations. Data extracted from each study included; study sample size, group descriptions, study design, analysis method, and outcome data. Furthermore, methodological quality was assessed using the modified 0–10 PEDro scale (de Morton, 2009). The primary outcome variables were defined as TT, bio-T, free-T, or sal-T pre- and post-intervention. Standard differences in means (SDM) were computed for the three meta-analyses by the software using the following equation (Higgins and Green, 2011):

$$\text{SDM}\;=\;(\mu_{1}\;-\;\mu_{2})÷\sigma$$

Whereby: μ_1_ = treatment mean, μ_2_ = control mean, and σ = pooled standard deviation

Where the SD for change between time points (i.e., pre- and post-training change) was not reported, it was calculated thusly:

$$\sigma_{\text{change}}\;=\;\sqrt{\left(\sigma_{1}^{2}+\sigma_{2}^{2}-\left(2\cdot corr\cdot \sigma_{1}\cdot \sigma_{2}\right)\right)}$$

Whereby: corr = correlation coefficient, a value that describes the relationship between baseline and final measurements over time. The correlation coefficient observed in our laboratory was 0.9 for TT pre-and post-intervention in a group of older males and therefore this was the value used for analysis.

Where a study utilized a UCT design (i.e., one group before and after training), the pre-training value was considered as μ_2_ and the post-training value was considered as μ_1_. Subgroup analyses were performed based on hormone fraction (i.e., TT, bio-T, free-T, or sal-T) where possible. Further analyses were performed based on research design as a means of investigating heterogeneous results.

## Results

### Study Selection

**Figure 1:**
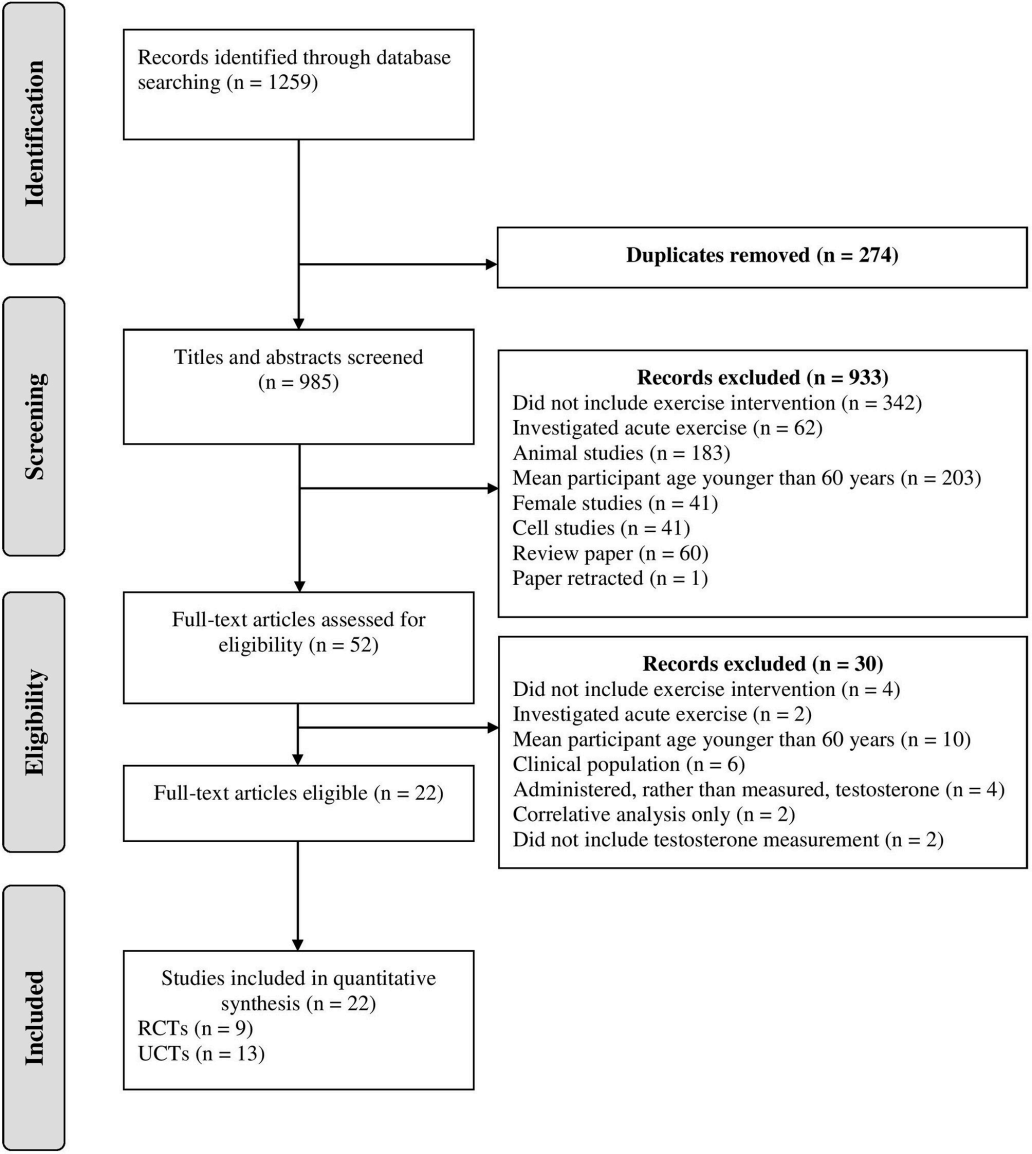
Schematic flow diagram describing exclusions of potential studies and final number of studies. RCT, randomized control trial; UCT, uncontrolled trial.

After the initial database search, 1259 records were identified (see Figure 1). Once duplicates were removed, 985 titles and abstracts were screened for inclusion by the authors resulting in 52 studies being retrieved as full text and assessed for eligibility. Of those, 30 were excluded and 22 articles remained and were used in the final quantitative synthesis. To assess publication bias, funnel plots for each exercise modality were computed and the trim and fill method was used (Duval and Tweedie, 2000). The trim and fill method determines the amount of studies required to eradicate publication bias from the funnel plot. For aerobic, resistance, and interval training, the resultant number of imputed studies to eradicate bias was 0.

### Study Characteristics

Of the 22 studies included, 9 were RCTs and 13 were UCTs (Tables 1–3). Where a study had multiple outcome measures (i.e., TT, bio-T, free-T, sal-T), they were treated as separate data points. Similarly, where a study reported values at multiple time points (i.e., 6 weeks, 12 weeks) they were treated separately.

**Table 1:** Description of included endurance training studies and data sets.

**References**	**Exercise intervention**	**Design method**	**Outcome measures**	**Participants**	**PEDro score**
Hayes et al., 2013b	Aerobic conditioning; 70–80% HR_max_, 150 min·wk^−1^, achieved by 2 d·wk^−1^ for 6 weeks	UCT vs. baseline	Salivary testosterone (ELISA)	*N* = 28 (age 63 ± 5, mass 90 ± 16 kg, stature 175 ± 3 cm). Lifelong sedentary.	3
Hayes et al., 2015d	Aerobic conditioning; 70–80% HR_max_, 150 min·wk^−1^, achieved by 2 d·wk^−1^ for 6 weeks	UCT vs. baseline	Total testosterone (ECLA) Bioavailable testosterone (Vermeulen equation) Free testosterone (Vermeulen equation)	*N* = 28 (age 63 ± 5, mass 90 ± 16 kg, stature 175 ± 3 cm). Lifelong sedentary.	3
Lovell et al., 2012	Periodized aerobic training; 2–3 d·wk^−1^ for 12 weeks.	RCT	Total testosterone (ECLA) Free testosterone (ECLA)	*N* = 12 (age 75 ± 3, mass 77 ± 12 kg, stature 177 ± 5 cm). Moderately active but without planned physical activity.	7
Struder et al., 1999	Periodized aerobic walking training; 5.6 km·h^−1^ for ~30–60 min; 3 d·wk^−1^ for 20 weeks.	RCT	Total testosterone (EIA) Free testosterone (RIA)	*N* = 11 (age 69 ± 3, mass 76 ± 11 kg, stature 174 ± 8 cm). Sedentary.	7

*HR_max_, maximum heart rate; RCT, randomized control trial; UCT, uncontrolled trial; ECLA, electrochemiluminescence assay; EIA, enzyme immunoassay; ELISA, enzyme-linked immunosorbent assay. RIA, radioimmunoassay*.

**Table 2:** Description of included resistance training studies and data sets.

**References**	**Exercise intervention**	**Design method**	**Outcome measures**	**Participants**	**PEDro score**
Ahtiainen et al., 2011	1. Periodized resistance training; multi-set, multi-exercise, 40–90% 1-RM, ~2 d·wk^−1^ for 10 weeks. 2. Periodized resistance training; multi-set, multi-exercise, 40–90% 1-RM, ~2 d·wk^−1^ for 21 weeks.	RCT	Total testosterone (ECLA)	*N* = 10 (age 61 ± 5, mass 80 ± 5 kg, stature 177 ± 3 cm). Healthy but untrained.	7
Ahtiainen et al., 2015	Periodized resistance training; multi-set, multi-exercise, 40–90% 1-RM, 2 d·wk^−1^ for 12 months.	UCT vs. baseline	Total testosterone (ECLA) Free testosterone (Vermeulen equation)	*N* = 9 (age 70 ± 2, mass 80 ± 8 kg). Physically active but not resistance trained.	3
Armamento-Villareal et al., 2016	1. Multi-component training for 90 min; Aerobic: 65–85% HR_max_, Resistance: multi-exercise, 65–85% 1-RM, 3 d·wk^−1^ for 6 months. 2. Multi-component training for 90 min; Aerobic: 65–85% HRmax, Resistance: multi-exercise, 65-85% 1-RM, 3 d·wk^−1^ for 12 months.	RCT	Total testosterone (automated immunoassay)	*N* = 10 (age 72 ± 2, mass 110 ± 3 kg). Frail, obese elderly.	6
Craig et al., 1989	Periodized resistance training; multi-set, multi-exercise, ~8–10-RM, 3 d·wk^−1^ for 12 weeks.	UCT vs. baseline	Total testosterone (RIA)	*N* = 9 (age 63 ± 1, mass 76 ± 2 kg). Physically active but not resistance trained.	3
Glintborg et al., 2013	1. Periodized resistance training; multi-set, multi-exercise, 2–3 d·wk^−1^ for 12 weeks. 2. Periodized resistance training; multi-set, multi-exercise, 2–3 d·wk^−1^ for 24 weeks.	RCT	Bioavailable testosterone (LC-MS/MS and Vermeulen equation)	*N* = 16 (age 68 [62–72]). Overweight men with low bioavailable testosterone. *N* = 9 (age 68 [62–72]). Overweight men with low bioavailable testosterone.	7
Glintborg et al., 2015	1. Progressive heavy strength training, 2–3 d·wk^−1^ for 3 months. 2. Progressive heavy strength training, 2–3 d·wk^−1^ for 6 months.	RCT	Bioavailable testosterone (LC-MS/MS and Vermeulen equation)	*N* = 9 (age 68 [62–72]). Overweight men with low bioavailable testosterone.	6
Hakkinen et al., 2002	1. Periodized heavy resistance training; multi-set, multi-exercise, 3-8-RM, 2 d·wk^−1^ for 12 weeks. 2. Periodized power training; multi-set, multi-exercise. 30–50% 1-RM, 2 d·wk^−1^ for 24 weeks.	RCT	Total testosterone (ELISA) Free testosterone (RIA)	*N* = 10 (age 65 ± 5, mass 84 ± 12 kg, stature 173 ± 7 cm). Healthy, mildly physically active.	7
Hakkinen and Pakarinen, 1994	Periodized resistance training; multi-set, multi-exercise, ~30–80% 1-RM, 2–3 d·wk^−1^ for 4 weeks.	UCT vs. baseline	Total testosterone (RIA) Free testosterone (RIA)	*N* = 10 (age 65 ± 1, mass 83 ± 6 kg, stature 171 ± 6 cm). Habitually active with no background in regular strength training.	3
	Periodized resistance training; multi-set, multi-exercise, ~30–80% 1-RM, 2–3 d·wk^−1^ for 8 weeks.			
	Periodized resistance training; multi-set, multi-exercise, ~30–80% 1-RM, 2–3 d·wk^−1^ for 12 weeks.			
Izquierdo et al., 2001	1. Periodized heavy and explosive resistance training; multi-set, multi-exercise, ~30–80% 1-RM. 2 d·wk^−1^ for 8 weeks. 2. Periodized heavy and explosive resistance training; multi-set, multi-exercise, ~30–80% 1-RM. 2 d·wk-1 for 16 weeks.	UCT vs. baseline	Total testosterone (RIA) Free testosterone (RIA)	*N* = 11 (age 64–73, mass 81 ± 10 kg, stature 167 ± 4 cm). Physically active but not resistance trained.	3
Katznelson et al., 2006	Theraband resistance training; multi-set, multi-exercise. 3–4 d·wk^−1^ for 12 weeks.	RCT	Total testosterone (RIA)	*N* = 15 (age 72 ± 6, mass 81 ± 14 kg).Ambulatory, community dwelling, sedentary men with serum free-testosterone < 14.5 pg·ml^−1^.	7
Kraemer et al., 1999	1. Periodized resistance training; multi-set, multi-exercise, 3-15-RM, 3 d·wk^−1^ for 3 weeks. 2. Periodized resistance training; multi-set, multi-exercise, 3-15-RM, 3 d·wk^−1^ for 6 weeks. 3. Periodized resistance training; multi-set, multi-exercise, 3-15-RM, 3 d·wk^−1^ for 10 weeks.	UCT vs. baseline	Total testosterone (RIA) Free testosterone (RIA)	*N* = 9 (age 62 ± 3, mass 84 ± 13 kg, stature 174 ± 7 cm). Physically active but not resistance trained.	3
Kvorning et al., 2013	1. Periodized resistance training; multi-set, multi-exercise, 6-20-RM, 3 d·wk^−1^ for 12 weeks. 2. Periodized resistance training; multi-set, multi-exercise, 6-20-RM, 3 d·wk^−1^ for 24 weeks.	RCT	Bioavailable testosterone (LC-MS/MS and Vermeulen equation)	*N* = 8 (age 70 ± 2, mass 91 ± 1 kg, stature 178 ± 2 cm). Overweight men with low bioavailable testosterone.	7
Lovell et al., 2012	Periodized resistance training; multi-set, multi-exercise, ~30–80% 1-RM, 2-3 d·wk^−1^ for 12 weeks.	RCT	Total testosterone (ECLA) Free testosterone (ECLA)	*N* = 12 (age 74 ± 3, mass 79 ± 14 kg, stature 178 ± 5 cm). Moderately active but without planned physical activity.	7
Petrella et al., 2006	Periodized resistance training; knee extensors, 8–12-RM, 3 d·wk^−1^ for 16 weeks.	UCT vs. baseline	Total testosterone (RIA) Free testosterone (Sodergard equation)	*N* = 13 (age 65 ± 1, mass 88 ± 3 kg, stature 179 ± 2 cm). Healthy but not resistance trained.	3
Sato et al., 2014	Periodized resistance training; knee extensors and flexors, 70% 1-RM, 3 d·wk^−1^ for 12 weeks.	UCT vs. baseline	Free testosterone (EIA)	*N* = 13 (age 67 ± 2, mass 64 ± 1 kg, stature 167 ± 1 cm). Moderately active but not resistance trained.	3
Vaczi et al., 2014	1. Periodized resistance training using eccentric contractions; knee extensors, 8–14 repetitions, 2-3 d·wk^−1^ for 10 weeks.	UCT vs. baseline	Total testosterone (ECLA)	*N* = 8 (age 64 ± 4, mass 78 ± 12 kg, stature 182 ± 9 cm). Recreationally active but not resistance trained.	3
	2. Periodized resistance training using stretch-shortening cycle; knee extensors, 8–14 repetitions, 2–3 d·wk^−1^ for 10 weeks.			*N* = 8 (age 66 ± 5, mass 79 ± 8 kg, stature 173 ± 6 cm). Recreationally active but not resistance trained.
Walker et al., 2015	Periodized resistance training; knee extensors and flexors, ~8-14-RM, 2 d·wk^−1^ for 20 weeks.	UCT vs. baseline	Total testosterone (ECLA)	*N* = 8 (age 64 ± 3, mass 84 ± 7 kg, stature 177 ± 5 cm). Physically active but not resistance trained.	3

*RM, repetition maximum; RCT, randomized control trial; UCT, uncontrolled trial; ECLA, electrochemiluminescence assay; RIA, radioimmunoassay; LC-MS/MS, liquid chromatography tandem mass spectrometry; ELISA, enzyme-linked immunosorbent assay; EIA, enzyme immunoassay*.

**Table 3:** Description of included interval training studies and data sets.

**References**	**Exercise intervention**	**Design method**	**Outcome measures**	**Participants**	**PEDro score**
Hayes et al., 2017a	1. Aerobic preconditioning at 70–80% HR_max_ followed by HIIT described below. 150 min·wk^−1^, achieved by 2 d·wk^−1^ for 6 weeks, then 6 weeks' HIIT 2. HIIT; 6 × 30 s @ 40% PPO, once every 5 days, for 6 weeks.	UCT vs. baseline UCT vs. preconditioning	Total testosterone (ECLA) Free testosterone (Vermeulen equation)	*N* = 22 (age 62 ± 2, mass 91 ± 16 kg, stature 175 ± 6 cm). Lifelong sedentary.	3
Herbert et al., 2017a	HIIT; 6 × 30 s @ 40% PPO, once every 5 days, for 6 weeks.	UCT vs. baseline	Total testosterone (ECLA) Free testosterone (Vermeulen equation)	*N* = 17 (age 60 ± 5, mass 78 ± 12 kg, stature 173 ± 6 cm). Masters athletes in endurance events.	3

*HIIT, high intensity interval training; HRmax, maximum heart rate; PPO, peak power output; UCT, uncontrolled trial; ECLA, electrochemiluminescence assay*.

#### Effect of Endurance Training on Testosterone

**Figure 2:**
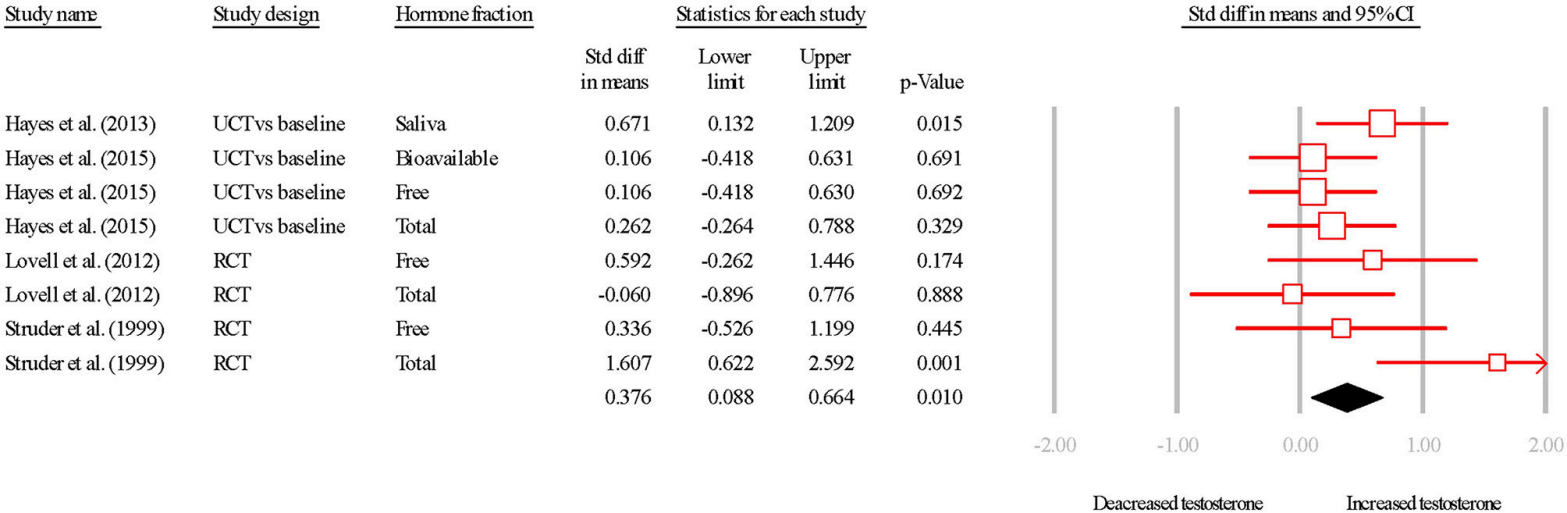
Summary of studies examining aerobic exercise interventions on testosterone concentrations. 1,2,3 indicate separate conditions within one investigation. Filled diamond indicates overall SDM. SDM, standard difference in means; RCT, randomized controlled trial; UCT vs. baseline, uncontrolled trial; pre-intervention compared to post-intervention testosterone. Note that symbol size of individual studies is representative of the weighting for the pooled SDM.

**Figure 3:**
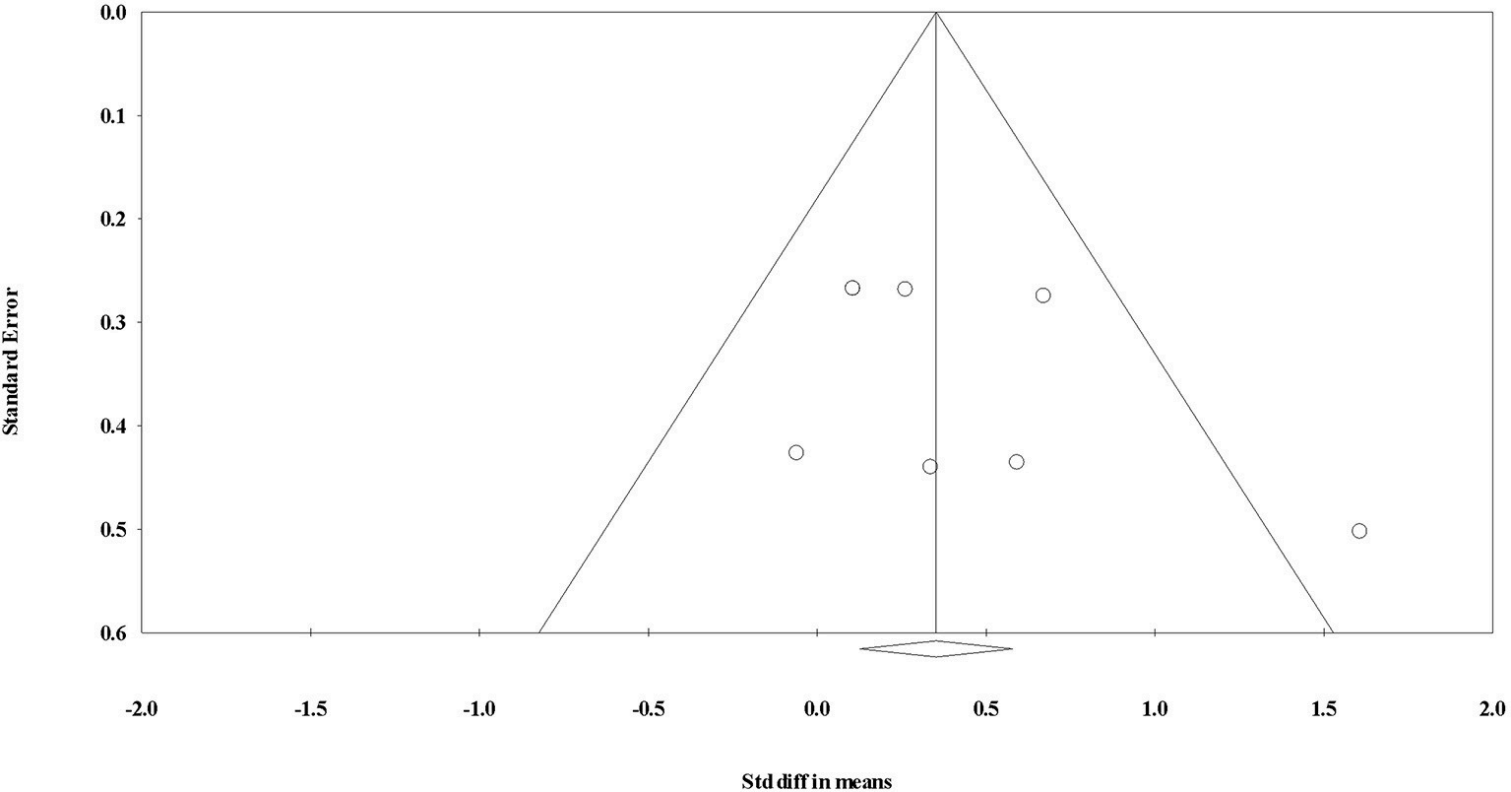
Funnel plot for evaluating the effect of aerobic exercise on testosterone concentrations.

The overall SDM of endurance training was 0.398 (95% CI = 0.034–0.761; *P* = 0.010; Figures 2, 3), and heterogeneity justified the use of a random effects model (*I*^2^ = 34.152). Due to the small number of studies, the effect of hormone fraction and study design was not tested.

#### Effect of Resistance Training on Testosterone

**Figure 4:**
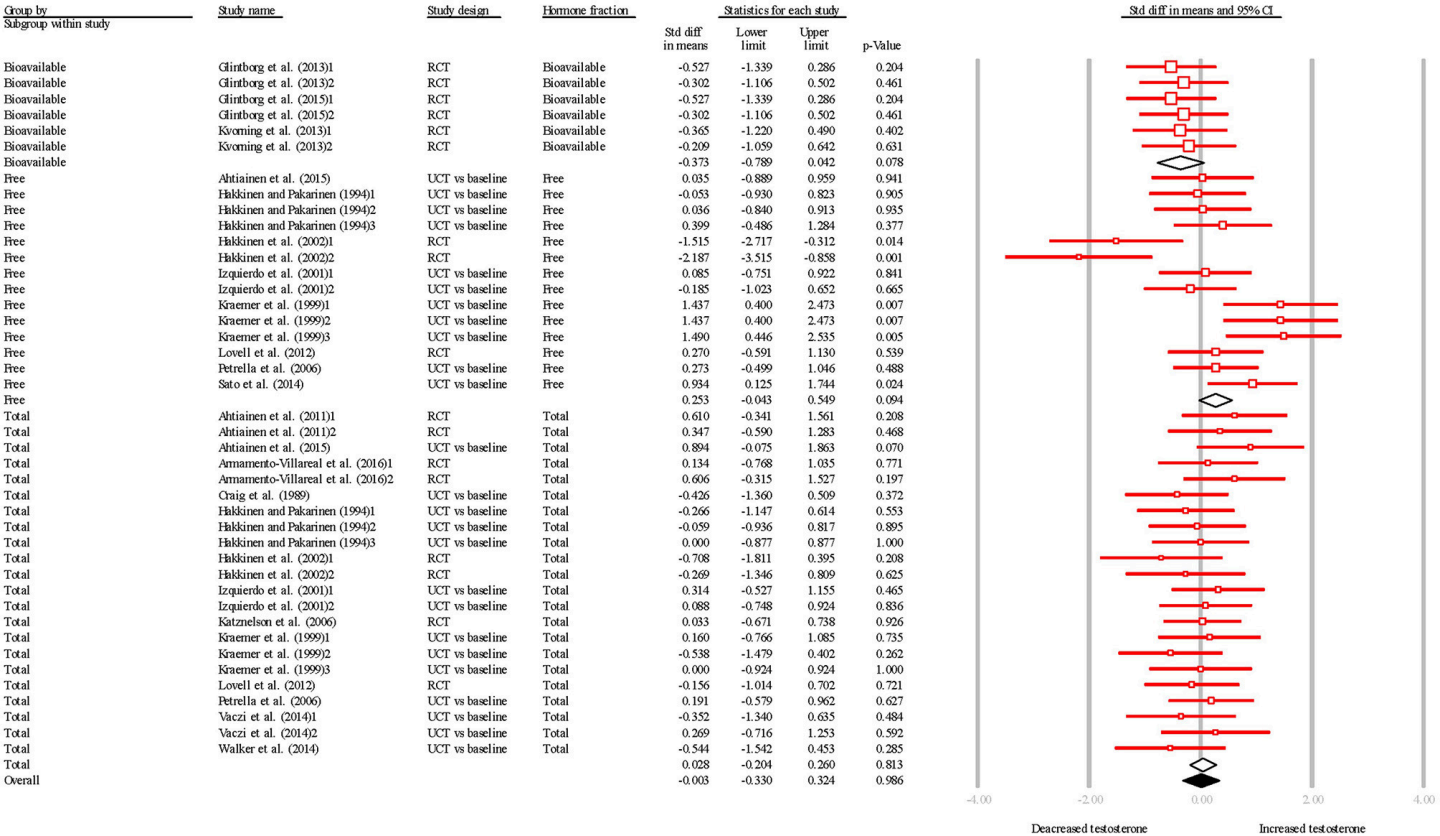
Summary of studies examining resistance exercise interventions on testosterone concentrations. 1,2,3 indicate separate conditions within one investigation. Filled diamond indicates overall SDM. Empty diamond indicates pooled SDM for the hormone fraction. SDM, standard difference in means; RCT, randomized controlled trial; UCT vs. baseline, uncontrolled trial; pre-intervention compared to post-intervention testosterone. Note that symbol size of individual studies is representative of the weighting for the pooled SDM.

**Figure 5:**
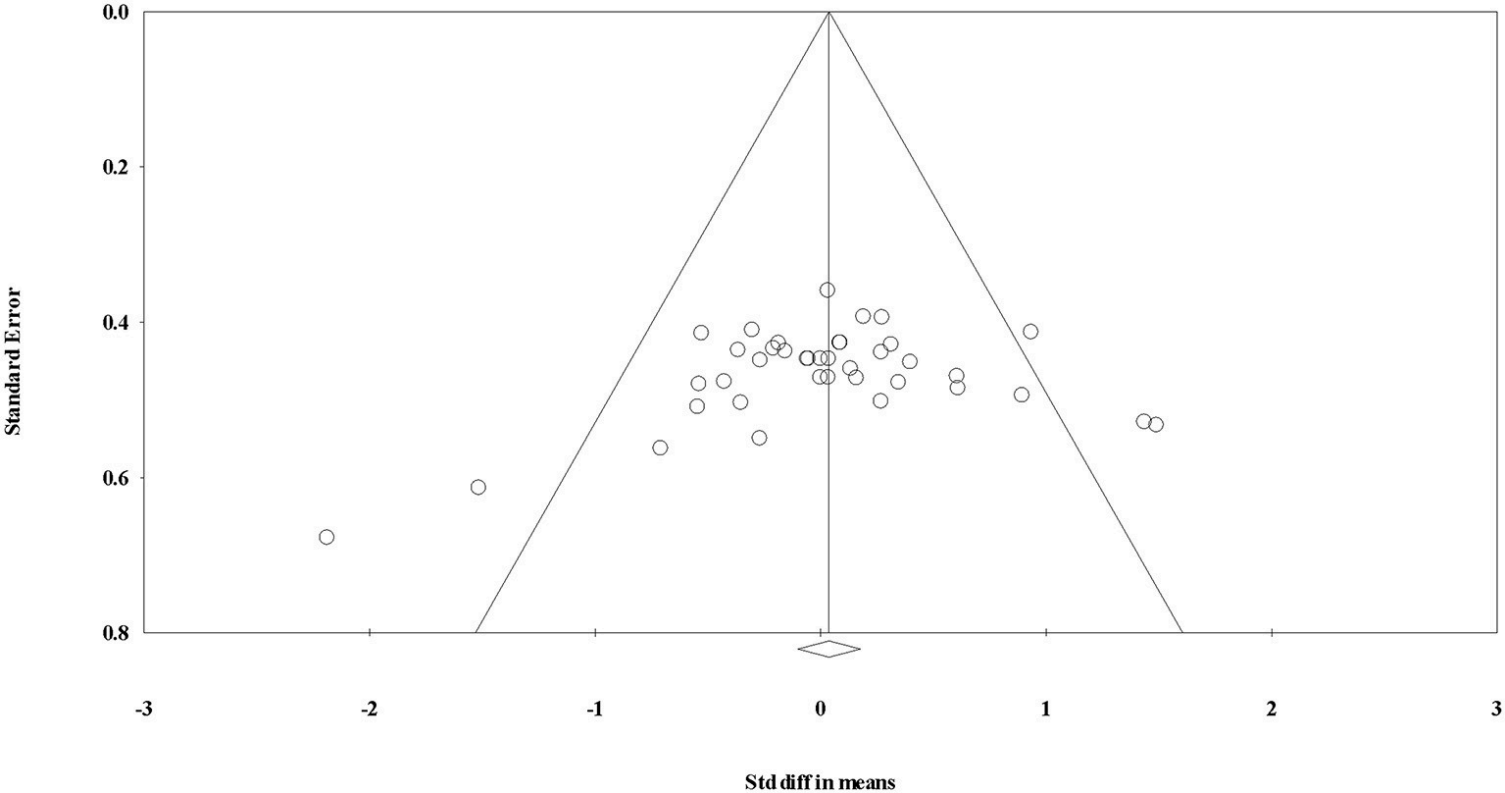
Funnel plot for evaluating the effect of resistance exercise on testosterone concentrations.

The overall SDM of resistance training was −0.003 (95% CI = −0.330–0.324; *P* = 0.986; Figures 4, 5) and heterogeneity justified the use of a random effects model (*I*^2^ = 37.340). Qualitative and quantitative effects of hormone fraction were observed, whereby free-T resulted in the greatest SDM (0.253; 95% CI = −0.043–0.549; *P* = 0.094), followed by TT (0.028; 95% CI = −0.204–0.260; *P* = 0.813), and bio-T (-0.373; 95% CI = −0.789–0.042; *P* = 0.631). There was a qualitative effect of study design (i.e., the direction of the effect was influenced) whereby UCTs resulted in a greater SDM (0.205; 95% CI = −0.011–0.421; *P* = 0.063), than RCTs (−0.218; 95% CI = -0.481–0.045; *P* = 0.105).

#### Effect of Interval Training on Testosterone

**Figure 6:**
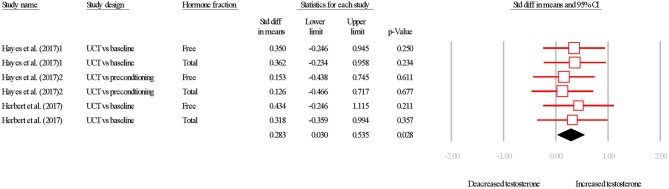
Summary of studies examining interval exercise interventions on testosterone concentrations. 1,2,3 indicate separate conditions within one investigation. Filled diamond indicates overall SDM. SDM, standard difference in means; UCT vs. baseline, uncontrolled trial; pre-intervention compared to post-intervention testosterone. UCT vs. preconditioning, uncontrolled trial; post-intervention testosterone was compared to after a phase of- “aerobic preconditioning.' Note that symbol size of individual studies is representative of the weighting for the pooled SDM.

**Figure 7:**
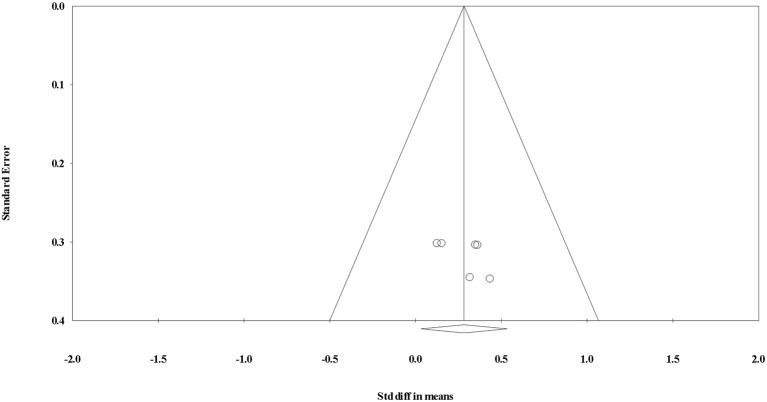
Funnel plot for evaluating the effect of interval exercise on testosterone concentrations.

The overall SDM of interval training was 0.283 (95% CI = 0.030–0.535; *P* = 0.028; Figures 6, 7) with heterogeneity observed as *I*^2^ = 0.000. Therefore, using a random or fixed effects model had no effect on the SDM or CIs, so for consistency we used a random effects model. Due to the small number of studies, the effect of hormone fraction and study design was not tested.

## Discussion

### Overall SDM

The main finding from this meta-analysis was that short-term exercise training inconsistently influences basal testosterone in older men. The magnitude and statistical significance of effect varied with exercise modality, study design, and hormone fraction. Given that exercise training has been proposed as a first-line treatment for mild age-associated testosterone decrements (Swerdloff and Anawalt, 2014), this meta-analysis provides timely insight into the effect of exercise interventions on basal testosterone.

### Endurance Training

The pooled effect of endurance training studies was a positive effect on basal testosterone in older males. Beside that of Lovell et al. (2012), studies displayed a positive SDM for endurance training. However, this finding of no change in response to training is a result of TT increasing similarly in the intervention group (~1.1 nmol·L^−1^) and in the control group (~1.1 nmol·L^−1^). What is interesting to note however, is the moderate increase in free-T in the same participants, following the same intervention. It would therefore seem reasonable to expect that a reduction in SHBG was responsible for this increased free fraction in the treatment group. In fact, SHBG increased less in the intervention group (~1.0 nmol·L^−1^) than in the control group (~2.5 nmol·L^−1^), which explains the moderate effect reported for free-T (treatment = ~0.8 pmol·L^−1^; control = ~-1.4 pmol·L^−1^). As such, it is postulated that biological or analytical variation likely caused this effect on free-T, which is supported by the large 95% CI. Similarly, Hayes et al. (2013b) reported a large increase in sal-T following aerobic conditioning, but sal-T is subject to large biological and analytical variation (Hayes et al., 2014), and this magnitude of effect was not the same in TT or free-T (Hayes et al., 2015d, 2017a).

### Resistance Training

When all studies were pooled, resistance training had no effect on basal testosterone in older males. The largest negative effect for free-T was observed by Hakkinen et al. (2002). When comparing to similar duration interventions (Ahtiainen et al., 2015), and similar resistance training programmes (Hakkinen and Pakarinen, 1994; Kraemer et al., 1999; Kvorning et al., 2013), it is difficult to explain these results merely with time course or training variables. Moreover, the study exhibiting the largest positive effect on free-T (Kraemer et al., 1999) used a similar resistance training programme as Hakkinen et al. (2002), and the same detection method (radioimmunoassay [RIA]). As such, we suggest there is little effect of resistance training on any testosterone fraction in the aging male. This suggestion is supported by the studies with the largest (Kraemer et al., 1999) and smallest (Hakkinen et al., 2002) SDM not achieving statistical significance. When examining the results of Hakkinen et al. (2002) more closely, we propose the negative effect on TT was primarily a result of a trivial change in the intervention group, exaggerated by a trivial change of the opposite direction in the control group. The training group of Hakkinen et al. (2002) experienced no change to TT, yet the control group experienced an increase from ~14.8 nmol·L^−1^ to ~15.9 nmol·L^−1^ which resulted in an SDM of −0.269 for the intervention group over 24 weeks. Moreover, the increase in the control group represents a change of ~13%, which is a trivial change using *post-hoc* analysis (Cohen's *d* = 0.18) and is within the critical difference outlined for TT (i.e., the threshold which needs to be exceeded for a change to be classed as biologically meaningful; Valero-Politi and Fuentes-Arderiu, 1993). Similarly, the training group experienced a ~0.3 pmol·L^−1^ reduction in free-T, whereas the control group experienced a ~14 pmol·L^−1^ increase, which resulted in an SDM of −2.187 over 24 weeks. However, the critical difference for free-T is yet to be established, and the increase of ~33% may not be biologically meaningful.

There were minor effects of sampling time on SDM. For example, Ahtiainen et al. (2011) observed a larger (yet still non-significant) change in free-T after 10 weeks, with a non-significant reduction from 10 weeks−21 weeks. An increase in androgen receptor (AR) expression could explain this minor decrease over time, as this would permit more testosterone-receptor interactions, which would remove free-T from circulation. However, no alteration to androgen receptor expression was observed by Ahtiainen et al. (2011). Further ambiguity is created by other studies reporting increased TT with longer time periods (Hakkinen et al., 2002). Again, we propose these changes are not biologically significant and are within the measurement error.

Whilst the effect of supraphysiological doses of testosterone on skeletal muscle are well-described (Bhasin et al., 2001; Sinha-Hikim et al., 2002; Deane et al., 2013; Hughes et al., 2016), the present investigation calls into question the importance of basal testosterone concentrations for increasing muscle mass in older men. Indeed, Vaczi et al. (2014) observed increased muscle strength and size despite clinically low testosterone in an older male population. Moreover, many investigations cited here reported increased muscle strength and size in the absence of increased basal testosterone (Hakkinen and Pakarinen, 1994; Izquierdo et al., 2001; Hakkinen et al., 2002; Ahtiainen et al., 2011).

Suppression of endogenous testosterone has been shown to attenuate muscle strength and mass gains in the young (Kvorning et al., 2006), and supraphysiological administration of testosterone increases muscle strength and mass (even without training) (Bhasin et al., 2001). As such, it appears that both low testosterone, and high testosterone, may be causal in muscular adaptations (or lack thereof). However, when testosterone is within a “normal” physiological range, neither the acute elevation (West et al., 2010), nor the basal concentration appear to drive resistance training adaptations in an older population. This is supported by individuals with the lowest testosterone in this meta-analysis still experiencing increased muscle strength and mass. Increased AR expression, or testosterone-AR binding affinity could provide a mechanistic link between the *in vitro* data demonstrating testosterone's trophic effect on muscle, and increased muscle size without a concomitant increase in testosterone. However, further experimental investigation is necessary to confirm these speculations.

### Interval Training

The two included interval training studies displayed a positive testosterone SDM, with both investigations coming from the same research group (Hayes et al., 2017a; Herbert et al., 2017a). This group used the same high intensity interval training (HIIT) in both studies, which consisted of 6 × 30 s sprints at 40% peak power output (~130% peak oxygen uptake) interspersed with 3 min rest. Interestingly, both sedentary older males and endurance-trained masters athletes experienced increased free-T. As the magnitude of change was similar for free-T and TT in both investigations, this increase in free-T was likely driven by TT, rather than reductions in SHBG. These data are encouraging as HIIT has been promoted as a time-efficient method to improve cardiometabolic health (Gillen and Gibala, 2014; Cassidy et al., 2017; Phillips et al., 2017), and any androgenic improvement would be an additional benefit. However, caution must be exerted when (a) drawing conclusion from only two studies from one laboratory, and (b) implementing HIIT in older adults (Riebe et al., 2015). As such, further evidence is required to determine if the testosterone response to HIIT is consistent in older males, and whether HIIT is tolerable and safe in older populations.

### Study Design

From the present meta-analysis, it is tempting to conclude RCTs result in different SDMs to UCTs. However, due to the distinct lack of RCTs it is difficult to justify such a conclusion, and further RCTs are warranted to add greater credence to the field. A further issue is the heterogeneity of exercise training design. When two studies used the same exercise intervention, for the same duration, free-T and TT responses were remarkably similar considering sedentary older men were compared to masters athletes (Hayes et al., 2017a; Herbert et al., 2017a). As such, it is possible the exercise intervention specifics (volume, intensity, frequency, etc.) may be more predictive of testosterone response to training that participant details or hormone fraction measured. However, without an investigation examining the testosterone response to two or more training programmes, matched for all variables except one (the dependent variable), this is purely speculation.

As with most exercise adaptations, individuals or groups with poorer starting values may be more susceptible to improvement. For example, untrained individuals commencing strength training should expect to increase their maximal strength more than experienced power lifters. Thus, it could be expected that individuals with low testosterone at enrolment would experience the greatest increase post-training. One investigation recruited individuals for their low testosterone (Kvorning et al., 2013), whilst three studies reported low mean starting TT < 12 nmol·L^−1^ (Vaczi et al., 2014; Ahtiainen et al., 2015; Armamento-Villareal et al., 2016), and all included a resistance training intervention. Kvorning et al. (2013) observed a negative SDM for bio-T in aging men with low-normal testosterone, whilst Ahtiainen et al. (2015) reported no change to free-T but a positive change to TT. Armamento-Villareal et al. (2016) reported a substantial change in TT after 12 months but not after 6 months. As such, investigations recruiting biochemically hypogonadal individuals also report inconsistent findings, thus starting testosterone concentrations appears unlikely to influence the response to resistance training. Finally, Vaczi et al. (2014) reported no change to TT in individuals with TT ~4 nmol·L^−1^. However, ~4 nmol·L^−1^ is in the very lowest range seen in our laboratory (Hayes et al., 2017a) and is classed as hypogonadal (Harman et al., 2001), and for that to be the mean value, an error in measurement or reporting may have occurred.

## Limitations

The major limitation of the present meta-analysis is the lack of included studies, especially in aerobic and intervals training models, therefore a greater number of investigations would add weight to conclusions made herein. As such, conclusions made here are conservative and preliminary, until a greater depth of literature is available concerning exercise and basal testosterone in older males. Whilst the literature assessment was comprehensive, it is possible that studies may have been missed from the analysis, but as three databases were searched, it is unlikely enough were missed to create a large change to SDMs. Furthermore, having two authors ensured agreement on inclusion and exclusion, which limited potential bias.

To reduce heterogeneity, studies were classified into one of three broad exercise categories reflecting the physiological requirements of each training type. Yet, volume, intensity, and frequency of training cannot be controlled for. In fact, often it is difficult to discern the above acute programme variables within each study due to vagaries in reporting. For example, some investigations report % one-repetition maximum, whereas some authors report a number repetition maximum. Similarly, rest periods and number of sets are rarely reported in resistance training studies. It would improve the literature base if all authors adhered to the consensus on exercise reporting template (CERT; Slade et al., 2016) in future investigations. Whilst some investigations included have achieved statistical significance, the change to be considered biologically significant remains to be fully elucidated. Therefore, it is difficult to ascertain whether responses are clinically meaningful. While meta-analyses describe a population effect, i.e., group mean change, no investigations have reported whether individuals cross a clinical threshold (i.e., from hypogonadal to eugonadal), and therefore exercise as a treatment for low testosterone cannot be prescribed with confidence.

## Conclusion

There is a pervasive belief that resistance exercise increases basal testosterone over time. However, that was not observed in the in older males in this meta-analysis. In fact, HIIT and endurance training showed the most promise for increasing basal testosterone in older men. There is a need for more RCTs to improve the quality of available evidence, as only seven studies in the present investigation achieved a score of 5 on the PEDro scale. The practical implication of this article is that resistance exercise may not be a viable solution to increase basal testosterone in the aging male, but aerobic and interval training may be. However, few studies examine whether exercise can raise testosterone from hypogonadal to eugonadal levels, which is of most clinical relevance and use for physicians.

Whilst here we report inconsistent effects of exercise training on basal testosterone in the eugonadal aging male, we do not argue that exercise training has a positive benefit in an aging population. Indeed, in many of the cited literature in this meta-analysis a lack of testosterone increase has not precluded physiological improvements following a training stimulus. Therefore, for older men, alterations in basal testosterone within the “normal” physiological range may not be mechanistically necessary for adaptation. Furthermore, testosterone is only one factor in the hormonal milieu, and exercise has been shown efficacious at improving other hormonal variables associated with successful aging and muscle function (Elliott et al., 2017; Sellami et al., 2017, 2018). Therefore, at present, exercise is probably our best non-pharmacological countermeasure to loss of muscle function with human aging.

## Author Contributions

LH and BE extracted and analyzed the data, drafted the manuscript, and proofed the manuscript.

### Conflict of Interest Statement

The authors declare that the research was conducted in the absence of any commercial or financial relationships that could be construed as a potential conflict of interest.
